# Identification of Normal Blood Pressure in Different Age Group: Erratum

**DOI:** 10.1097/MD.0000000000010685

**Published:** 2018-05-04

In the article, “Identification of Normal Blood Pressure in Different Age Group”,^[1]^ which appeared in Volume 95, Issue 14 of *Medicine*, the affiliation for Jiunn-Diann Lin and Chung-Ze Wu should be:

Division of Endocrinology, Department of Internal Medicine, Shuang-Ho Hospital, Taipei Medical University, New Taipei City, Taiwan; Division of Endocrinology and Metabolism, Department of Internal Medicine, School of Medicine, College of Medicine, Taipei Medical University, Taipei, Taiwan.

## References

[1] Lin J-D Chen Y-L Wu C-Z Identification of Normal Blood Pressure in Different Age Group. *Medicine*. 2016 95:e3188. 27057846 10.1097/MD.0000000000003188 PMC4998762

